# Analysis of the efficiency and influencing factors of the urban and rural residents social endowment insurance in China: from the perspective of regional heterogeneity

**DOI:** 10.3389/fpubh.2025.1560163

**Published:** 2025-04-02

**Authors:** Aibi Dai, Yuanbo Hu, Henglang Xie, Wei Zhao, Burak Keskin

**Affiliations:** 1 School of Smart Health and Wellness, Zhejiang Dongfang Polytechnic, Wenzhou, China; 2 School of Digital Business, Zhejiang Dongfang Polytechnic, Wenzhou, China; 3 School of Business and Economics, Universiti Putra Malaysia, Serdang, Malaysia; 4 Department of High-tech Business and Entrepreneurship, Faculty of Behavioural, Management and Social Sciences, University of Twente, Enschede, Netherlands; 5 Faculty of Economics and Administrative Sciences, Cankiri Karatekin University Uluyazi Campus, Cankiri, Türkiye

**Keywords:** urban and rural residents social endowment insurance, operational efficiency, DEA-Tobit model, regional heterogeneity, population aging

## Abstract

**Introduction:**

China’s urban and rural residents social endowment insurance (URRSEI) plays a key role in ensuring the quality of life of the older adult and promoting social stability.

**Methods:**

Based on the data of 31 provinces in China from 2012 to 2022, this study evaluates the efficiency of URRSEI and analyzes its influencing factors through DEA method and Tobit Model.

**Results and discussion:**

The overall efficiency of China is high. Eastern region has the highest efficiency. The efficiency of central region is relatively stable. Western region has the lowest average efficiency. The level of local fiscal social security expenditure is negatively correlated with efficiency, while the increase of institutional maintenance rate and educational resources significantly improves the performance of URRSEI. The urban registered unemployment rate and the number of unemployment insurance recipients play a positive role in the security function.

## 1 Introduction

China’s public pension is an important source of basic livelihood for the older adult population. The pension insurance fund is not only an important tool to ensure the basic life of the older adult, but also the core pillar to promote social stability and economic development. With the aging of the population and the decline of the birth rate, pension insurance funds play a key role in narrowing the gap between the rich and the poor and improving people’s livelihood. Urban and rural residents social endowment insurance is an important part of China’s social insurance system, which provides a key guarantee for urban and rural residents who lack old-age insurance (1), and plays an active role in reducing poverty among older adults and coping with future uncertainty (2, 3). The Chinese government has successively promulgated the Guiding Opinions on Launching the Pilot Program of New Rural Social Pension Insurance and the Opinions on Establishing a Unified Basic URRSEI (4). In the context of the aging of the population, improving the operational efficiency of URRSEI can maintain social stability, promote the rational distribution of resources among different groups, and achieve sustainable economic and social development (5). China’s URRSEI and pension insurance system for urban workers constitute a dual-track design to meet the needs of different regions and groups of people, and to ensure fairness and coverage (1).

However, the differences in policies, participation rates and payment standards between provinces have led to large regional differences in the operational efficiency of URRSEI (6, 7). In highly urbanized areas, there is often a more diversified economic structure with a larger proportion of formal-sector employment. This leads to a more stable income source for residents, enabling them to contribute more steadily to the URRSEI. Also, urban areas usually have more developed financial and administrative systems, which can facilitate the management and operation of the pension system, thus enhancing its efficiency. Regions with an aging population may face challenges in maintaining high URRSEI efficiency. A larger older adult population means a higher demand for pension payouts. If the proportion of the working—age population contributing to the system is relatively small, it may strain the financial sustainability of the URRSEI, resulting in lower efficiency. In contrast, regions with a younger population structure may have a more favorable contribution—to—payout ratio, potentially leading to higher efficiency.

There are significant differences between China and foreign countries in terms of system design and operational efficiency of pension insurance funds. In the face of the pressure of aging, pension insurance systems of the United Kingdom, the United States and other countries have adopted two main reform measures. One is to adjust the existing system to balance income and expenditure by reducing pensions or extending the calculation period and delaying retirement. The other is the capitalization transformation, which promotes the implementation of private pension schemes and supports a hybrid model through state guarantees and tax incentives (8). The efficient management and long-term investment capacity of the fund can not only alleviate fiscal pressures on governments, but also have a positive impact on economic growth and social cohesion by promoting long-term savings, promoting employment, and enhancing personal financial responsibility (5). As an important pillar of the welfare state, public pension insurance funds ensure the financial security of the older adult group after retirement and reduce the risk of poverty (9). At the same time, the optimization of fund governance and investment strategies is crucial to improving operational efficiency. The United Kingdom and the United States maintain pension systems through a pay-as-you-go model, but their effectiveness relies on the premise that population growth is higher than interest rates (10). Compared with these countries, the management of China’s pension insurance fund faces greater challenges in cross-regional and cross-system coordination. These regional differences provide a practical basis for examining the operational efficiency of URRSEI from a regional perspective. China needs to explore a more flexible reform path to ensure the long-term sustainability of pension funds, promote policy innovation and optimize fund management. While ensuring fairness, the efficient operation of the fund can further enhance the social security function and provide a solid foundation for economic transformation and social progress (2, 8). Therefore, it is necessary to evaluate the operational efficiency of URRSEI in China (11). How to accurately assess the efficiency of its operation has become a very important issue.

This study not only evaluates the operational efficiency of URRSEI in China, but also analyzes the efficiency of the eastern, central, and western regions, and analyzes the factors that affect its efficiency. The contributions of this paper are reflected in the following aspects. First, this study systematically reveals how the economic development level and social security policies in different regions shape the operational efficiency of URRSEI, which provides a new perspective for understanding the efficiency differences between provinces. Second, this study explores the role of multiple factors such as fiscal expenditure, social security, and employment level on the operational efficiency of URRSEI, which not only enriches the theoretical basis for the evaluation of old-age insurance efficiency, but also provides methodological support for future research. Third, this paper highlights the profound impact of regional heterogeneity on institutional operation, especially the contrast between economic flexibility in the eastern region and policy bottlenecks in the western region, and deepens the understanding of governance efficiency in different regions. Finally, the policy recommendations put forward have strong practical value, which can provide a scientific basis for optimizing policy support and improving the sustainability of URRSEI.

The remaining sections are arranged as follows. Section 2 presents the literature on the efficiency and influencing factors of URRSEI. Section 3 describes the research methodology and data sources. Section 4 presents the empirical research results, analyzes the results and comparative analysis of URRSEI, and the influencing factors. Section 5 provides conclusions and policy implications.

## 2 Literature review

### 2.1 Efficiency evaluation of URRSEI

With the changes in the global economic and social environment, the operational efficiency of the pension system has gradually become an important indicator to measure the effectiveness of the pension system in various countries (12). While traditionally more focused on the adequacy dimension, operational efficiency has become increasingly important across countries and regions. The efficiency of pension funds in different countries is also different, and the pension systems of European countries show a divergent trend in terms of management efficiency. Hungary, Luxembourg and Romania lead the way in pension fund efficiency, while Greece, Portugal and Italy are less efficient (9). There are significant differences in efficiency between countries in terms of gross domestic product (GDP) allocation, adequacy, and labor market impact (9). In 2017–2020, some pension funds in Greece performed well, but efficiency differences within the industry remained significant (13). The pension system in the Netherlands exhibits significant administrative economies of scale, but some diseconomies of scale in investment management (3). A study on the efficiency of pension fund management in Kenya revealed that the middle-aged group had the best governance quality of funds (2). Australia’s superannuation funds have been less efficient than expected in spending, but risk management has performed well (14). The sustainability of Ukraine’s pension system improved during the period 2016–2020, but it was still not enough to effectively prevent poverty in old age and replace retirement income (15). Research on supplementary pension in Poland noted that the performance of high-fee products was broadly in line with the market average, although the investment efficiency of individual retirement products showed relatively stable returns (16). Russia’s pension system is too reliant on investments in debt instruments, resulting in inadequate returns and difficulty in coping with inflation (17).

There are significant differences in the operational efficiency of the pension system between different regions. Although the overall operational efficiency of China’s pension insurance is at a high level, there are significant differences in performance between regions (1). Economically developed regions do not necessarily have higher pension performance, and there is not a complete coordination between the level of economic development and pension insurance performance in each province (18). The rural–urban divide is also an important issue, with the rural population’s pension insurance participation rate much lower than that of the urban population, especially among migrant workers in the construction industry, many of whom are unable to participate in urban pension schemes due to policy inflexibility (19, 20). The impact of pension on the older adult aged 60–69 years, those with low educational attainment, and the older adult in the eastern region is more significant (21). In addition, the coverage and development level of pension insurance between regions also showed a trend of “high in the east and low in the west,” and the coordination between the economic development level and pension insurance in each province was insufficient (18). There is a significant gap in the operational efficiency of URRSEI in different regions, which reveals the constraints of the lag in policy formulation on the coordinated development of the region. The empirical analysis therefore focuses on how fiscal input, demographic pressure, and regional development conditions are associated with URRSEI efficiency. From 2016 to 2020, the performance of China’s urban workers’ pension insurance showed a downward trend in various provinces, and regional development was uneven and uncoordinated (18). The existence of such regional differences means that to improve the efficiency of URRSEI, it is necessary to optimize the policy design and promote the coordinated development of various regions.

### 2.2 Factors affecting the operational efficiency of URRSEI

The operational efficiency of URRSEI is affected by multiple factors, including enterprise investment, fiscal policy, economic environment and system design. There is a significant negative correlation between the proportion of pension insurance contributions and the investment efficiency of enterprises, and higher contribution rates will crowd out investment opportunities of enterprises (22, 23). Data from listed companies in China show that reducing the proportion of pension contributions can significantly increase total factor productivity, especially among non-state-owned, small and medium-sized enterprises, and labor-intensive enterprises (24). From a macro point of view, there is an inverted U-shaped relationship between the old-age dependency ratio and the efficiency of pension fund collection. When the dependency ratio is too high, the efficiency of fund collection decreases, reflecting the dynamic balance between the dependency burden and the efficiency of the system (25). The younger the official is in his current position, the more obvious the inverted U-shaped relationship between the tenure of the official and the governance efficiency of the basic pension insurance (11). At the regional level, GDP, urbanization level and the scale of government public expenditure have a positive impact on the operational efficiency of basic pension insurance, but the old-age dependency ratio has a significant negative effect on it (1). The management structure and size of pension funds also affect their operational efficiency. Studies in India have found that public sector funds are generally more efficient than private sector funds, and that fund size is positively correlated with efficiency (26). The case of Taiwan shows that ownership structure has a significant impact on investment performance, and optimal management can help achieve long-term stable returns for funds (27). In the study of dynamic efficiency in European countries, the improvement of technological efficiency and institutional reform have played a key role in the improvement of social security efficiency (28). In addition, the level of corporate pension funds is closely related to market returns. Underfunded firms have instead achieved higher returns on the stock market (29). The trade-off between fairness and efficiency in the design of pension systems is also crucial (30). Pension increases in closed economies weaken welfare levels, while in the context of capital flows, pension increases are likely to benefit only capital-exporting countries (31). Fiscal pressures caused by the financial crisis and poor pension performance have prompted many countries to adjust their contribution policies, while China has responded to the challenges of aging by delaying retirement (5).

The government’s financial investment in pensions is not simply linearly related to the efficiency of the pension system. In China, the level of local fiscal social security expenditure is negatively correlated with the efficiency of social pension insurance for urban and rural residents. This may be due to the lack of effective supervision over the use of fiscal funds, resulting in resource waste and affecting the efficiency of the pension system. In some European and American countries, reasonable fiscal investment has improved the social equity and efficiency of the pension system by subsidizing low-income groups and other means (32). The investment in educational resources has a positive impact on the efficiency of the pension system. A good level of education can help increase workers’ income, thereby increasing pension contributions, and also enhance public awareness and participation in the pension system. Social expenditures such as healthcare are also related to the efficiency of the pension system. A healthy older adult population can reduce the pressure of pension expenditures and indirectly improve the sustainability and efficiency of the pension system (33). Therefore, to understand the influencing factors of the operational efficiency of URRSEI, it is necessary to comprehensively consider the complex factors at the micro and macro levels to balance the relationship between enterprise development, financial sustainability and social welfare.

The existing research on URRSEI in China has accumulated important results in terms of system value, operational efficiency, influencing factors and regional differences, but there is still room for further deepening. First of all, most studies focus on the analysis of a certain period of time, and the trend of pension insurance efficiency in the long-term dynamic change is not fully revealed. Secondly, although the effects of old-age dependency ratio, fiscal expenditure and urbanization level on efficiency have been explored in previous studies, there is still a lack of systematic analysis of the complex interaction between different factors, especially the linkage effect between local fiscal expenditure and employment indicators needs to be further explored. In addition, the research on the urban–rural gap mainly focuses on the low participation rate of rural areas, but how to improve the participation enthusiasm of disadvantaged groups and the pertinence of policies still need to be further explored. In terms of regional heterogeneity, although the existing studies have revealed the efficiency distribution pattern of “high in the east and low in the west,” the analysis of the underlying economic and social factors is not systematic enough. At the same time, the impact of important reforms such as the delayed retirement policy on institutional efficiency has not been fully verified, especially the lack of empirical support for the specific effects among different regions.

## 3 Methodology

Data envelopment analysis (DEA) is a widely used method for assessing non-parametric efficiency, which was first proposed by Charnes, Cooper, and Rhodes (34). It is suitable for evaluating the relative efficiency of decision-making units (DMUs) with multiple inputs and outputs, and has been widely used in evaluating the efficiency of enterprise innovation, transportation systems, environmental performance, and energy quota trading (35–38). Simar and Wilson (39) emphasized the importance of using DEA to analyze the efficiency of pension funds’ investment management. By considering inputs such as management costs, staff resources, and outputs like investment returns and pension benefits, DEA can provide a comprehensive evaluation of the efficiency of different pension—related entities (40) used DEA to analyze the efficiency of pension systems in EU countries. Inputs included the proportion of GDP spent on pensions, labor force participation rates, and outputs were pension replacement rates and poverty rates among the older adult. Their research revealed that there were significant variations in the efficiency of pension systems among EU member states, with some countries achieving higher efficiency in converting inputs into desired outputs.

The basic DEA model is shown in Equation (1).


$$\begin{array}{l} \min\theta -\varepsilon \sum (s^{-}+s^{+})\\ s.t.\{\begin{array}{l} \sum_{j=1}^{n}\text{λjxij}+s_{i}^{-}=\text{θxik}\\ \sum_{j=1}^{n}\text{λjyrj}-s_{i}^{+}=\mathrm{yrk}\\ \sum_{j=1}^{n}\mathrm{\lambda j}=1\\ \mathrm{\lambda j}\geq 0;s^{-}\geq 0;s^{+}\geq 0\\ j=1,2,\cdots ,n\\ i=1,2,\cdots ,m;r=1,2,\cdots ,q \end{array} \end{array}$$ (1)

Banker, Charnes and Cooper proposed the BCC model (41). This model allows the DUMs to perform efficiency analysis under the assumption of variable returns to scale, which solves the limitation of the constant returns to scale. The number of DMUs is *n*. *j* represents the *j*th DMU. *λ_j_* represents the weight of the DMU reference set*. x* and *y* represent the variables of inputs and outputs, with the number of *m* and *q*, respectively. *i* and *r* represent the *i*-th input variable and the *r*-output variable, respectively. *θ* represents the relative efficiency. *s^+^* represents the relaxation variable of output. *s^−^* represents the relaxation variable of input.

In this paper, two key input indicators are chosen to comprehensively assess the URRSEI system. The income of URRSEI is selected as one of the input indicators. The income of the fund serves as a fundamental measure of the economic foundation and the extent of financial support for URRSEI. It is used to quantify the influx of funds into the system. A higher income indicates a more substantial financial base, which can potentially support better pension provisions and system operations. For example, if the income mainly comes from contributions by insured individuals and government subsidies, a larger income implies more resources available for paying out pensions in the future. Without sufficient income, the system may struggle to meet its long-term obligations, making it a crucial indicator for understanding the system’s financial viability. The number of people insured by URRSEI is the second input indicator. This reflects the participation level of residents in the URRSEI program. A larger number of insured people not only broadens the financial base through contributions but also indicates a higher degree of social coverage. When more people are insured, it implies that the system is fulfilling its social security function more effectively, reaching a wider segment of the population. This is significant as the goal of URRSEI is to provide a safety net for as many urban and rural residents as possible. If the number of insured people is declining, it may signal potential problems such as low awareness of the program, unaffordable contribution levels, or lack of trust in the system (42).

For the output indicators, three aspects are considered. The cumulative balance of URRSEI is selected to evaluate the long-term financial health of the fund. It represents the net result of contributions and expenditures over time. A healthy cumulative balance indicates that the system has been able to manage its finances well, with contributions exceeding expenditures in a sustainable manner. This is important because a positive cumulative balance ensures the long—term solvency of the system. If the cumulative balance is negative or decreasing rapidly, it may suggest that the system is paying out more than it is receiving, which could lead to financial instability in the long run. The expenditure of the social endowment insurance fund for urban and rural residents is another output indicator. Fund expenditures are key to assessing the efficiency and quality of the fund’s use and management. Higher expenditures may not necessarily mean better performance. Instead, it is important to ensure that the funds are being used effectively to provide adequate pension benefits. For instance, if the expenditure is mainly focused on administrative costs rather than pension payouts, it may indicate inefficiencies in the system. Moreover, fund expenditures are crucial for evaluating the burden of the system. If the expenditures are growing at an unsustainable rate, it may put pressure on the financial sustainability of the URRSEI, potentially leading to the need for increased contributions or reduced benefits in the future. Finally, the actual number of people receiving URRSEI is chosen as an output indicator. This number directly shows the number of residents who are actually benefiting from the urban and rural housing security benefits. It is a clear measure of the system’s effectiveness in fulfilling its core function of providing social security. A higher number of beneficiaries means that more people are being supported by the system, which is a positive sign of the system’s success. Conversely, a low number of beneficiaries relative to the number of insured may indicate problems such as strict eligibility criteria, complex claim processes, or insufficient benefit levels that are deterring people from accessing the benefits they are entitled to (43, 44).


$$y_{\mathrm{it}}=\{\begin{array}{l} {y_{\mathrm{it}}}^{*}=\beta x_{\mathrm{it}}+\varepsilon_{\mathrm{it}},y>0\\ 0,y_{\mathrm{it}}\leq 0 \end{array}$$ (2)

The efficiency values evaluated by the DEA method range from 0 to 1 are border-truncated, which makes the traditional ordinary least squares method have estimation biases. The Tobit model is suitable for the data with the dependent variable truncated within a certain interval and can effectively estimate the linear relationship of the truncated variable (45), as shown in Equation 2. *β* is the parameter to be evaluated. *x_it_* is the independent variable. y_it*_ is the dependent variable. *y_it_* is the efficiency value variable, that is, the operating efficiency of URRSEI.

This paper selects the level of local fiscal social security expenditure (LFSSE), the system maintenance rate (DRIS), the urban registered unemployment rate (URUR), the year-end unemployment insurance benefit (UIRY), and the number of students enrolled in higher educations (SUN) as the factors of the operational efficiency of URRSEI. The level of LFSSE can measure the impact of government support on urban and rural housing security funds (43). If a region has a DRIS, it usually faces higher spending pressures and needs to improve management efficiency to cope with the imbalance between fund income and expenditure (44). URUR represents how healthy the job market is. The level of unemployment will directly affect the income stability and social security needs of residents, which will have a significant impact on the payment pressure and operational efficiency of URRSEI (46). The number of people receiving UIRY indicates whether the unemployed are able to receive timely support in times of economic hardship. This is not only related to the coverage of short-term social security, but also indirectly reflects the resilience of the pension system to cope with long-term financial needs. The number of students enrolled in higher educations (SUN) not only indicates the level of educational development, but also reveals the potential of the future labor market, influencing the size of the social insurance contributors.

Data for the variables were obtained from the National Bureau of Statistics of China. For some missing values, the mean interpolation method was used to deal with them. In addition, all explanatory variables were standardized to eliminate the influence of dimensions and avoid the effects of extreme values.

## 4 Results

### 4.1 Efficiency evaluation

Based on panel data from 31 provincial-level administrative regions in China from 2012 to 2022, this study evaluates the operational efficiency of URRSEI. The results are shown in Table 1. From 2012 to 2022, the efficiency level of URRSEI in 31 provincial-level administrative regions across the country showed significant time changes and regional differences. The efficiency levels of the provinces also fluctuate from year to year. Beijing’s efficiency was low at the beginning of 2012, only 0.5211, but gradually showed an upward trend, increasing to 0.9942 in 2022, which indicates that Beijing has made positive adjustments in the optimization of pension insurance policies and resource allocation. It is also worth noting that although the Tibet started late, with an efficiency of only 0.5191 in 2012, it continued to improve in the following decade, reaching 0.7317 in 2022, showing its gradual progress in the field of old-age security. However, some regions, such as Jilin and Inner Mongolia, have experienced a decline in efficiency in some years, indicating that policy implementation and fund management still need to be further optimized. For example, the average efficiency of Guizhou and Guangxi is close to or exceeds the national average, which is 0.7364 and 0.7889, respectively, reflecting the importance of policy support and management innovation.

**Table 1:** Evaluation results of URRSEI in provinces.

Provinces	2012	2013	2014	2015	2016	2017	2018	2019	2020	2021	2022	Mean
Shanghai	1	1	1	0.9989	0.9600	0.9997	0.9481	1	0.9568	0.9942	1	0.9048
Yunnan	0.6450	0.6554	0.7160	0.7332	0.7765	0.7985	0.8467	0.8701	0.5966	0.9233	1	0.7134
Inner Mongolia	0.6664	0.6509	0.7850	0.8210	0.7938	0.7523	0.8480	0.8354	0.7300	0.7263	0.7861	0.6996
Beijing	0.5211	0.5261	0.5254	0.6425	0.7937	0.8183	0.8371	0.8563	0.9036	0.9924	1	0.7014
Jilin	0.9746	0.9583	0.9834	0.9124	1	0.9352	0.9322	0.9215	0.8702	0.7852	0.8542	0.8439
Sichuan	1	1	1	0.9901	1	0.9661	0.9967	1	0.9041	0.8699	0.8836	0.8842
Tianjin	1	1	0.9850	0.9601	0.9799	0.9447	0.9652	0.9708	0.9819	1	1	0.8990
Ningxia	0.9001	0.8234	0.8038	0.8042	0.7617	0.7754	0.7462	0.7198	0.6291	0.5802	0.6402	0.6820
Anhui	0.8356	0.8457	0.9054	0.8853	0.9041	0.9204	0.8273	0.8407	0.8320	0.8318	0.7954	0.7853
Shandong	1	1	0.9599	1	0.9980	1	1	1	0.9833	0.9680	1	0.9091
Shanxi	0.6410	0.6458	0.6655	0.7181	0.7431	0.7518	0.7847	0.7843	0.7883	0.7608	0.7283	0.6676
Guangdong	0.8903	0.8751	0.7895	0.8398	0.9594	1	1	0.9484	1	0.9784	0.9167	0.8498
Guangxi	0.8770	1	0.8864	0.8519	0.8643	0.8459	0.8917	0.8399	0.7656	0.7958	0.8479	0.7889
Xinjiang	0.5533	0.5656	0.5840	0.5888	0.5782	0.5371	0.5156	0.4954	0.5162	0.5413	0.5842	0.5050
Jiangsu	1	0.9797	1	0.9865	0.9984	1	1	1	0.9799	0.9844	1	0.9107
Jiangxi	0.6592	1	0.7421	0.7624	0.7483	0.7357	0.7344	0.8124	0.7595	0.8056	0.8484	0.7173
Hebei	0.8310	0.8776	0.9502	0.9097	0.9478	0.9412	0.9282	0.8927	0.9000	0.8887	0.9973	0.8387
Henan	1	1	1	0.9904	1	1	0.9826	0.9907	0.9851	0.9892	0.9933	0.9109
Zhejiang	1	0.9737	0.9899	0.9882	0.9830	1	0.9911	1	0.9158	0.9725	1	0.9012
Hainan	0.8766	0.8294	0.7695	0.6900	0.5994	0.6388	0.5903	0.5752	0.6337	0.5851	0.6485	0.6197
Hubei	0.7409	0.8040	0.8547	0.8582	0.8584	0.8463	0.8266	0.8150	0.8290	0.8495	0.8084	0.7576
Hunan	0.9015	0.9094	0.9464	0.9062	0.9264	0.8941	0.9237	0.8727	0.9058	0.8198	0.8494	0.8213
Gansu	0.6325	0.6423	0.6621	0.6984	0.7061	0.6869	0.6852	0.7145	0.6946	0.7291	0.8332	0.6404
Fujian	0.7090	0.7172	0.7486	0.7743	0.7999	0.8326	0.8448	0.8203	0.8107	0.8386	0.8624	0.7299
Tibet	0.5191	1	0.7898	0.7869	0.7095	0.4916	0.6915	0.6857	0.6097	0.5967	0.6746	0.7138
Guizhou	0.8796	0.8014	0.7702	0.7863	0.7959	0.7835	0.8264	0.8505	0.8103	0.7480	0.7849	0.7364
Liaoning	0.9240	0.9253	0.9307	0.9354	0.9622	0.9609	0.9854	0.9682	0.9632	0.9924	0.9962	0.8786
Chongqing	0.7864	0.8286	0.8637	0.9498	0.9027	0.8257	0.8376	0.7979	0.8135	0.8028	0.7454	0.7629
Shaanxi	0.6536	0.6874	0.7267	0.7663	0.8082	0.8336	0.8240	0.8223	0.8187	0.8462	0.8988	0.7238
Qinghai	0.7426	0.7509	0.6642	0.6490	0.6591	0.6291	0.6319	0.5768	0.4459	0.6206	0.5472	0.5764
Heilongjiang	0.7010	0.8332	0.8693	0.8999	1	0.8027	0.8460	0.7777	0.7385	0.7073	0.7346	0.7425

**Figure 1:**
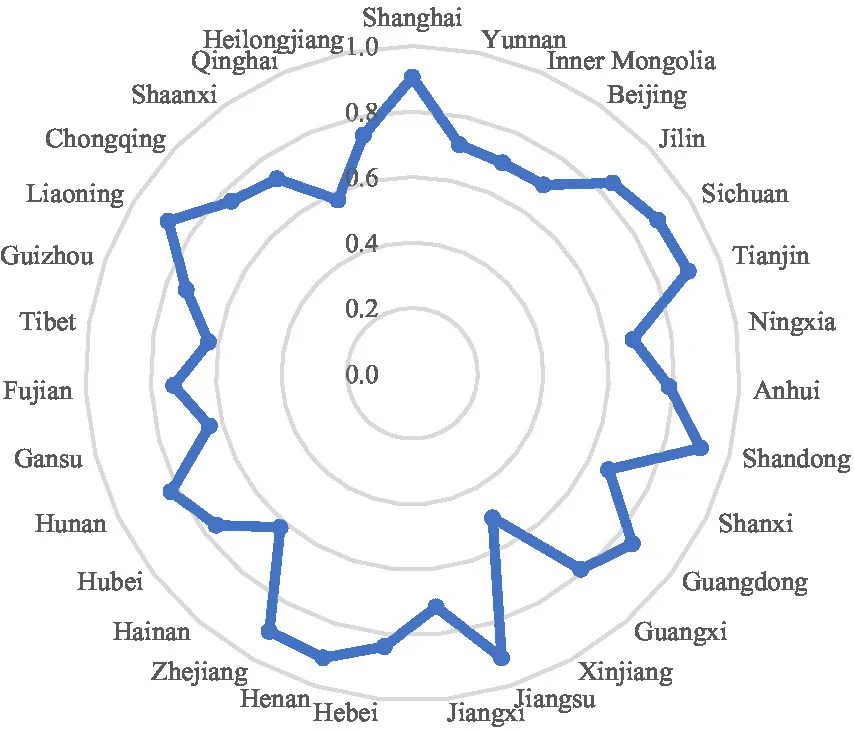
Average efficiency of 31 provinces.

Figure 1 shows the average efficiency of the 31 provinces. The distribution pattern of e efficiency in different regions can be observed more clearly. Economically developed regions such as Shanghai, Zhejiang, and Jiangsu have also shown high efficiency. Shanghai has maintained a stable high efficiency in each year, and the efficiency value has reached 1 in many years, reflecting Shanghai’s excellent performance in the management of URRSEI and the use of funds. The average efficiency of Zhejiang reached 0.9012, showing the superiority of the region in terms of pension fund management, coverage of insured persons and actual payment of benefits. In contrast, the average efficiency of economically backward areas such as Qinghai and Xinjiang is low, which is 0.5794 and 0.5884, respectively, indicating that there are still difficulties in the management and policy implementation of URRSEI, and there are many challenges in the management of resources, policy implementation and insurance coverage in these areas. Although Chongqing and Hunan provinces are not typical developed coastal areas, the efficiency values of their URRSEIs reach 0.7629 and 0.8213 respectively, which indicates that the local government has taken effective measures in the management of pension insurance. In addition, from a regional perspective, the eastern regions such as Zhejiang and Guangdong are not only economically developed, but also have advantages in the operation efficiency of pension insurance, which is closely related to their perfect social security system. However, the efficiency of provinces in the central and western regions, such as Shaanxi and Gansu, is relatively lagging behind, reflecting that there is still a gap between economic conditions and policy implementation. There is significant heterogeneity in the social URRSEI in China among different regions.

**Figure 2:**
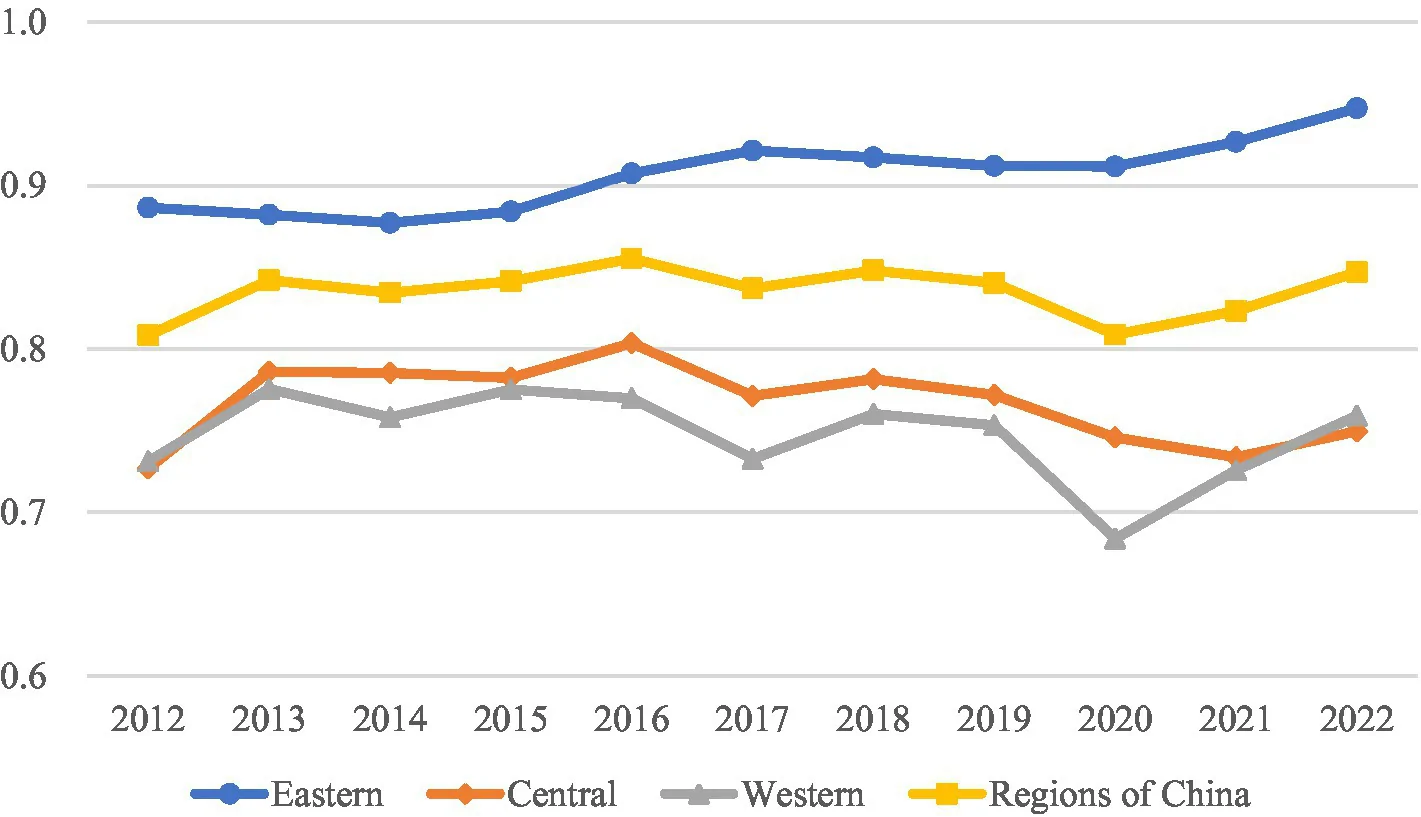
Regional innovation efficiency.

In order to further explore the differences between regions, the efficiency of each region was calculated, and the results are shown in Table 2 and Figure 2. Overall, the average efficiency of China is 0.7655, indicating that there is still room for further improvement in the operation of URRSEI across the country. The eastern region performed the most prominently in terms of overall efficiency, with an average value of 0.8312. The eastern region has always maintained a high efficiency for many years, which shows that the eastern region has a strong support capacity in the implementation of URRSEI due to its high level of economic development and sufficient financial resources. Its efficient operation is not only reflected in the rational management of fund expenditures and balances, but also reflects the effective coverage of the number of insured people and the number of people receiving benefits. The efficiency performance in the central region was relatively stable, with an average value of 0.7032. The central region reached a peak of 0.7861 in 2013 but has since fallen back to below 0.74, especially during 2020 and 2021. This trend may reflect the impact of the economic shock caused by the COVID-19 epidemic on fiscal spending and social security management capacity in the central region. Despite this, the central region has maintained a relatively balanced operational efficiency despite limited resources, showing the efforts and progress in policy implementation. The mean value in the western region was the lowest, only 0.6854, and showed large volatility during the sample period. This reflects the challenges faced by the western region in the implementation of URRSEI, including the problems of insufficient financial input and a relatively weak economic foundation. However, it is worth noting that the efficiency of the western region has increased to 0.7592 in 2022, indicating that with the increase of policy support and the continuous improvement of the social security system, and the western region is gradually improving the operation efficiency of its pension insurance. The operational efficiency of URRSEI in the eastern, central and western regions shows significant regional differences. The performance of different regions in terms of resource utilization, policy implementation and institutional guarantee directly affects their respective efficiency levels and stability.

**Table 2:** Evaluation results of URRSEI in different regions.

Region	2012	2013	2014	2015	2016	2017	2018	2019	2020	2021	2022	Mean
Eastern	0.8865	0.8822	0.8772	0.8841	0.9074	0.9215	0.9173	0.9120	0.9117	0.9268	0.9474	0.8312
Central	0.7270	0.7861	0.7853	0.7824	0.8035	0.7713	0.7816	0.7719	0.7458	0.7338	0.7496	0.7032
Western	0.7312	0.7755	0.7581	0.7753	0.7698	0.7327	0.7602	0.7533	0.6839	0.7258	0.7592	0.6854
China	0.8084	0.8421	0.8344	0.8414	0.8554	0.8370	0.8480	0.8405	0.8088	0.8234	0.8471	0.7655

### 4.2 Analysis of influencing factors

Based on the results of the evaluation of URRSEI, its influencing factors are further analyzed. The results are shown in Table 3. The variance inflation factor (VIF) values of the factors are less than 5, indicating that the collinearity problem between the variables is low, and regression analysis can be carried out.

**Table 3:** VIF results.

Variables	VIF	1/VIF
DRIS	2.25	0.44
SUN	2.14	0.47
LFSSE	2	0.5
UIRY	1.81	0.55
URUR	1.16	0.86
Mean VIF	1.87	

The results of influencing factor analysis are shown in Table 4. There are also regional differences in the impact of different factors on URRSEI. On a national scale, LFSSE has a significant negative impact on URRSEI (*p* < 0.01), indicating that the increase in fiscal expenditure may not directly improve the efficiency of URRSEI, and there may be problems such as unreasonable resource allocation or increased management costs. The impact of the DRIS on efficiency was significant positive (*p* < 0.05), indicating that a higher DRIS could improve the performance of social pension insurance. URUR and UIRY also have a significant positive impact on efficiency, indicating that the social security function of URRSEI is more fully reflected when the unemployment level is high. In addition, SUN also had a positive effect on the efficiency of the system (*p* < 0.01).

**Table 4:** Regression results.

Variables	China	Eastern	Central	Western
LFSSE	−0.0264***	−0.00345	−0.0701***	−0.0565***
	(0.00875)	(0.0151)	(0.0152)	(0.0205)
DRIS	0.0250**	0.0164	0.0930***	0.0303
	(0.0128)	(0.023)	(0.0251)	(0.0257)
URUR	0.0218***	0.0354***	−0.0028	0.0148
	(0.00635)	(0.0118)	(0.00941)	(0.0116)
UIRY	0.0466***	0.0257	0.0617	0.0497**
	(0.0109)	(0.0169)	(0.0481)	(0.0202)
SUN	0.0440***	0.038	0.0496**	0.0962***
	(0.0142)	(0.0234)	(0.0229)	(0.0308)
Constant	0.843***	0.912***	0.830***	0.813***
	(0.0142)	(0.0258)	(0.0285)	(0.0269)
LR test	chi-bar^2^ = 123.58, *p* < 0.01	chi-bar^2^ = 22.95, *p* < 0.01	chi-bar^2^ = 33.02, *p* < 0.01	chi-bar^2^ = 22.51, *p* < 0.01
rho	0.4923	0.4040	0.5601	0.3981
Wald χ^2^ test	Wald *χ*^2^ = 61.50, *p* < 0.01	Wald *χ*^2^ = 24.62, *p* < 0.01	Wald *χ*^2^ = 29.61, *p* < 0.01	Wald *χ*^2^ = 34.11, *p* < 0.01
Observations	341	121	110	110

Standard errors in parentheses. ***p < 0.01, **p < 0.05.

The regression results in the eastern region show that LFSSE has no significant negative impact on efficiency, indicating that eastern region has a low dependence on the improvement of the operation efficiency of pension insurance due to its developed economy. However, URUR has a significant positive impact on efficiency (*p* < 0.01), indicating that the security function of URRSEI was fully exerted when the unemployment rate increased, which may be related to the perfect social security system and high fiscal flexibility.

The regression results in the central region showed that LFSSE has a significant negative impact on operational efficiency (*p* < 0.01). This reflects that while the level of fiscal expenditure increases, there may be bottlenecks in management efficiency, resulting in the failure of resource input to be effectively translated into institutional performance. The impact of DRIS on efficiency is positive and significant (*p* < 0.01), indicating that the improvement of DRIS is helpful to enhance the operational effectiveness of URRSEI. The positive effect of SUN on efficiency is also significant (*p* < 0.01), which indicates that the social security system in the central region can operate more efficiently in the context of improving education level, providing support for the sustainability of the system in the future.

The regression results in the western region showed that LFSSE also has a significant negative impact on efficiency (*p* < 0.01), indicating that the increase of fiscal expenditure failed to effectively improve the performance of URRSEI, which may be due to insufficient resource management or certain obstacles to policy implementation in western region. UIRY has a significant positive impact on efficiency (*p* < 0.05), reflecting the important role of unemployment insurance in the social security system in the region. SUN plays a particularly prominent role in promoting efficiency (*p* < 0.01), indicating that the improvement of educational resources provides strong support for the operation of URRSEI in western region and helps to alleviate the pressure of population aging.

On the whole, the operational efficiency of URRSEI in different regions is affected by a variety of factors, and shows significant regional heterogeneity. The Wald *χ*^2^ test results showed that all regression models were statistically significant (*p* < 0.01), which verified the common effect of the selected explanatory variables on the operating efficiency. The likelihood ratio (LR) test results further show that the random-effects model is reasonable, and the heterogeneity between regions has an important impact on the performance of URRSEI. The national rho value is 0.4923, indicating that about 49.23% of the total variance comes from the heterogeneity between regions, reflecting the significant differences in the efficiency of institutional operation in different regions.

### 4.3 Robust test

In order to test the robustness of the regression results, the regression analysis was performed again after excluding the non-significant factors. The results are shown in Table 5. The comparison of the two regression results showed that there was no significant change in the coefficients and significances of each influencing factor. The significant influencing factors were still significant, and even the significances of some factors were improved. This indicates that our regression analysis results have good robustness.

**Table 5:** Robustness test results.

Variables	China		Eastern		Central		Western	
LFSSE	−0.0264***	−0.0264***	−0.00345		−0.0701***	−0.0699***	−0.0565***	−0.0642***
	(0.00875)	(0.00875)	(0.0151)		(0.0152)	(0.0142)	(0.0205)	(0.02)
DRIS	0.0250**	0.0250**	0.0164		0.0930***	0.0882***	0.0303	
	(0.0128)	(0.0128)	(0.023)		(0.0251)	(0.0249)	(0.0257)	
URUR	0.0218***	0.0218***	0.0354***	0.0352***	−0.0028		0.0148	
	(0.00635)	(0.00635)	(0.0118)	(0.012)	(0.00941)		(0.0116)	
UIRY	0.0466***	0.0466***	0.0257		0.0617		0.0497**	0.0609***
	(0.0109)	(0.0109)	(0.0169)		(0.0481)		(0.0202)	(0.0197)
SUN	0.0440***	0.0440***	0.038		0.0496**	0.0524**	0.0962***	0.0971***
	(0.0142)	(0.0142)	(0.0234)		(0.0229)	(0.0231)	(0.0308)	(0.0305)
Constant	0.843***	0.843***	0.912***	0.931***	0.830***	0.812***	0.813***	0.820***
	(0.0142)	(0.0142)	(0.0258)	(0.033)	(0.0285)	(0.0273)	(0.0269)	(0.0294)
LR test	chi-bar^2^ = 123.58, *p* < 0.01	chi-bar^2^ = 123.58, *p* < 0.01	chi-bar^2^ = 22.95, *p* < 0.01	chi-bar^2^ = 78.77, *p* < 0.01	chi-bar^2^ = 33.02, *p* < 0.01	chi-bar^2^ = 46.26, *p* < 0.01	chi-bar^2^ = 22.51, *p* < 0.01	chi-bar^2^ = 35.04, *p* < 0.01
rho	0.4923	0.4923	0.4040	0.6207	0.5601	0.6281	0.3981	0.4629
Wald χ^2^ test	Wald *χ*^2^ = 61.50, *p* < 0.01	Wald *χ*^2^ = 61.50, *p* < 0.01	Wald *χ*^2^ = 24.62, *p* < 0.01	Wald *χ*^2^ = 8.61, *p* < 0.01	Wald *χ*^2^ = 29.61, *p* < 0.01	Wald *χ*^2^ = 27.50, *p* < 0.01	Wald *χ*^2^ = 34.11, *p* < 0.01	Wald *χ*^2^ = 27.08, *p* < 0.01
Observations	341	341	121	121	110	110	110	110

Standard errors in parentheses. ***p < 0.01, **p < 0.05.

## 5 Discussion

### 5.1 Contribution to theory

This study contributes significantly to the theoretical understanding of social security systems, particularly in the context of China’s URRSEI. By evaluating the efficiency of URRSEI across 31 provinces from 2012 to 2022, it provides insights into regional disparities in social security effectiveness. The findings reveal that while the eastern region exhibits the highest efficiency, the western region has the lowest average efficiency. This highlights the importance of regional characteristics in shaping the performance of social security systems. When compared with European and American countries, where social security systems are often more centralized and funded by higher levels of public expenditure, China’s regional variation suggests a need for tailored approaches rather than one-size-fits-all solutions. Moreover, the negative correlation between local fiscal social security expenditure levels and efficiency challenges the conventional wisdom that higher public spending necessarily leads to better outcomes. Instead, it underscores the need for efficient resource allocation strategies. Additionally, the positive impact of institutional maintenance rates and educational resources on URRSEI performance emphasizes the interplay between education and social security, contributing new dimensions to existing theories.

### 5.2 Contribution to practice

From a practical standpoint, this research offers valuable empirical evidence for policymakers aiming to enhance the sustainability of URRSEI. The detailed analysis of regional efficiencies helps local governments identify strengths and weaknesses within their social security frameworks, enabling them to implement targeted improvements. For instance, the finding that higher local fiscal social security expenditures do not necessarily correlate with greater efficiency suggests that policy adjustments should focus on optimizing resource use rather than merely increasing funding. In contrast, many Western countries have established robust mechanisms for continuous evaluation and adjustment of social security policies based on comprehensive data collection and analysis. Furthermore, the significant role played by institutional maintenance rates and educational resources indicates that investments in these areas could yield substantial benefits for social security performance. Policymakers are advised to prioritize these aspects to strengthen the overall efficacy of the URRSEI system. Lastly, the positive influence of urban registered unemployment rates and the number of unemployment insurance recipients on social security functions suggests that employment policies can have complementary effects on social welfare, providing a basis for integrated policy-making.

### 5.3 Limitations

Despite its contributions, this study has several limitations. Firstly, reliance on official statistical data may introduce issues related to data quality and completeness, especially in less economically developed regions where data collection standards may vary. These inconsistencies could affect the accuracy of efficiency assessments and, consequently, the validity of policy recommendations derived from the findings. Secondly, while the quantitative approach used in this study effectively identifies correlations between variables, it falls short in exploring deeper causal relationships. Qualitative factors such as cultural contexts and variations in policy implementation at the local level are not fully accounted for, limiting the depth of analysis. Additionally, the study’s timeframe means that short-term socioeconomic fluctuations might not be fully captured, introducing uncertainty into long-term trend predictions. Future research should aim to incorporate more diverse data sources and employ mixed-method approaches to address these limitations, thereby providing a more comprehensive understanding of URRSEI dynamics.

## 6 Conclusion and policy implications

Based on the panel data of 31 Chinese provinces from 2012 to 2022, this study systematically evaluates the operational efficiency of URRSEI and reveals the heterogeneous performance of influencing factors in different regions. The results show that the average efficiency of China is 0.7655, indicating that there is still room for further optimization of China’s URRSEI. The eastern region has the highest overall efficiency. The average efficiency value of the central region is 0.7032. The average efficiency in the western region is the lowest. Nationwide, LFSSE has a significant negative impact on the operational efficiency of URRSEI. DRIS shows a positive effect on the whole country. There are significant differences in the influencing factors of efficiency in different regions. The role of URUR in the eastern region is more significant in terms of efficiency improvement, while the impact of LFSSE is not significant. The negative impact of LFSSE in the central region is significant, and the positive effect of SUN is prominent. The western region shows a stronger positive impact of UIRY.

Based on the empirical findings, policymakers should implement the following targeted recommendations to enhance URRSEI efficiency. Regional governments must prioritize resource allocation and management capacity-building in western provinces through inter-regional cooperation, leveraging eastern China’s high-efficiency models to establish mentorship programs. The Ministry of Finance needs to optimize fiscal expenditure structures by reducing redundant social security spending annually and redirecting saved funds toward digitalized pension management systems, ensuring 90% coverage of grassroots service networks in rural counties. Social security administrations are required to institutionalize maintenance rate improvements through quarterly system audits and performance-linked funding mechanisms. Education authorities should integrate educational resource development with pension literacy campaigns, launching mandatory public workshops in 15,000 townships by 2027 to boost contribution compliance rates. Cross-departmental task forces must establish real-time coordination between unemployment insurance and pension systems, utilizing AI-powered unemployment data analytics to dynamically adjust coverage for 8–10 million vulnerable urban migrants annually. The State Council should complement these measures with differentiated regional policies, such as offering tax incentives for western provinces achieving 80% efficiency benchmarks by 2028 while preserving central China’s stability through fixed fiscal transfer mechanisms.

## Data Availability

The data analyzed in this study is subject to the following licenses/restrictions: data will be made available on request. Requests to access these datasets should be directed to Yuanbo Hu, yuanbohu0110@163.com.
