# Correction: Analysis of the efficiency and influencing factors of the urban and rural residents social endowment insurance in China: from the perspective of regional heterogeneity

**DOI:** 10.3389/fpubh.2026.1926699

**Published:** 2026-08-12

**Authors:** Aibi Dai, Yuanbo Hu, Henglang Xie, Wei Zhao, Burak Keskin

**Affiliations:** 1 School of Smart Health and Wellness, Zhejiang Dongfang Polytechnic, Wenzhou, China; 2 School of Digital Business, Zhejiang Dongfang Polytechnic, Wenzhou, China; 3 School of Business and Economics, Universiti Putra Malaysia, Serdang, Malaysia; 4 Department of High-tech Business and Entrepreneurship, Faculty of Behavioural, Management and Social Sciences, University of Twente, Enschede, Netherlands; 5 Faculty of Economics and Administrative Sciences, Cankiri Karatekin University Uluyazi Campus, Cankiri, Türkiye

**Keywords:** urban and rural residents social endowment insurance, operational efficiency, DEA-Tobit model, regional heterogeneity, population aging

There was a mistake in Table 4 as published. Typographical errors introduced negative signs before several standard errors in parentheses. The corrected Table 4 appears below.

**Table 4:** Regression results.

**Variables**	**China**	**Eastern**	**Central**	**Western**
LFSSE	−0.0264^***^	−0.00345	−0.0701^***^	−0.0565^***^
	(0.00875)	(0.0151)	(0.0152)	(0.0205)
DRIS	0.0250^**^	0.0164	0.0930^***^	0.0303
	(0.0128)	(0.023)	(0.0251)	(0.0257)
URUR	0.0218^***^	0.0354^***^	−0.0028	0.0148
	(0.00635)	(0.0118)	(0.00941)	(0.0116)
UIRY	0.0466^***^	0.0257	0.0617	0.0497^**^
	(0.0109)	(0.0169)	(0.0481)	(0.0202)
SUN	0.0440^***^	0.038	0.0496^**^	0.0962^***^
	(0.0142)	(0.0234)	(0.0229)	(0.0308)
Constant	0.843^***^	0.912^***^	0.830^***^	0.813^***^
	(0.0142)	(0.0258)	(0.0285)	(0.0269)
LR test	chi-bar^2^ = 123.58, *p* < 0.01	chi-bar^2^ = 22.95, *p* < 0.01	chi-bar^2^ = 33.02, *p* < 0.01	chi-bar^2^ = 22.51, *p* < 0.01
rho	0.4923	0.4040	0.5601	0.3981
Wald χ^2^ test	Wald χ^2^ = 61.50, *p* < 0.01	Wald χ^2^ = 24.62, *p* < 0.01	Wald χ^2^ = 29.61, *p* < 0.01	Wald χ^2^ = 34.11, *p* < 0.01
Observations	341	121	110	110

Standard errors in parentheses. ^***^ *p* < 0.01, ^**^ *p* < 0.05.

There was a mistake in Table 5 as published. Typographical errors introduced negative signs before several standard errors in parentheses. Additionally, the corresponding model fit statistics (rho, LR test, and Wald χ^2^) in the three re-estimated models have been updated to their correct values. The corrected Table 5 appears below.

**Table 5:** Robustness test results.

**Variables**	**China**		**Eastern**		**Central**		**Western**	
LFSSE	−0.0264^***^	−0.0264^***^	−0.00345		−0.0701^***^	−0.0699^***^	−0.0565^***^	−0.0642^***^
	(0.00875)	(0.00875)	(0.0151)		(0.0152)	(0.0142)	(0.0205)	(0.02)
DRIS	0.0250^**^	0.0250^**^	0.0164		0.0930^***^	0.0882^***^	0.0303	
	(0.0128)	(0.0128)	(0.023)		(0.0251)	(0.0249)	(0.0257)	
URUR	0.0218^***^	0.0218^***^	0.0354^***^	0.0352^***^	−0.0028		0.0148	
	(0.00635)	(0.00635)	(0.0118)	(0.012)	(0.00941)		(0.0116)	
UIRY	0.0466^***^	0.0466^***^	0.0257		0.0617		0.0497^**^	0.0609^***^
	(0.0109)	(0.0109)	(0.0169)		(0.0481)		(0.0202)	(0.0197)
SUN	0.0440^***^	0.0440^***^	0.038		0.0496^**^	0.0524^**^	0.0962^***^	0.0971^***^
	(0.0142)	(0.0142)	(0.0234)		(0.0229)	(0.0231)	(0.0308)	(0.0305)
Constant	0.843^***^	0.843^***^	0.912^***^	0.931^***^	0.830^***^	0.812^***^	0.813^***^	0.820^***^
	(0.0142)	(0.0142)	(0.0258)	(0.033)	(0.0285)	(0.0273)	(0.0269)	(0.0294)
LR test	chi-bar^2^ = 123.58, *p* < 0.01	chi-bar^2^ = 123.58, s *p* < 0.01	chi-bar^2^ = 22.95, *p* < 0.01	chi-bar^2^ = 78.77, *p* < 0.01	chi-bar^2^ = 33.02, *p* < 0.01	chi-bar^2^ = 46.26, *p* < 0.01	chi-bar^2^ = 22.51, *p* < 0.01	chi-bar^2^ = 35.04, *p* < 0.01
rho	0.4923	0.4923	0.4040	0.6207	0.5601	0.6281	0.3981	0.4629
Wald χ^2^ test	Wald χ^2^ = 61.50, *p* < 0.01	Wald χ^2^ = 61.50, *p* < 0.01	Wald χ^2^ = 24.62, *p* < 0.01	Wald χ^2^ = 8.61, *p* < 0.01	Wald χ^2^ = 29.61, *p* < 0.01	Wald χ^2^ = 27.50, *p* < 0.01	Wald χ^2^ = 34.11, *p* < 0.01	Wald χ^2^ = 27.08, *p* < ado0.01
Observations	341	341	121	121	110	110	110	110

Standard errors in parentheses. ^***^ *p* < 0.01, ^**^ *p* < 0.05.

In the published article, Reference 4 and Reference 12 were cited in the literature discussion. Due to concerns raised about the cited papers, Reference 4 and Reference 12 and their associated text have been removed.

A correction has been made to the section **1 Introduction**, Paragraph 1:

“China's public pension is an important source of basic livelihood for the older adult population. The pension insurance fund is not only an important tool to ensure the basic life of the older adult, but also the core pillar to promote social stability and economic development. With the aging of the population and the decline of the birth rate, pension insurance funds play a key role in narrowing the gap between the rich and the poor and improving people's livelihood. Urban and rural residents social endowment insurance is an important part of China's social insurance system, which provides a key guarantee for urban and rural residents who lack old-age insurance (1), and plays an active role in reducing poverty among older adults and coping with future uncertainty (2, 3). The Chinese government has successively promulgated the Guiding Opinions on Launching the Pilot Program of New Rural Social Pension Insurance and the Opinions on Establishing a Unified Basic URRSEI (4). In the context of the aging of the population, improving the operational efficiency of URRSEI can maintain social stability, promote the rational distribution of resources among different groups, and achieve sustainable economic and social development (5). China's URRSEI and pension insurance system for urban workers constitute a dual-track design to meet the needs of different regions and groups of people, and to ensure fairness and coverage (1).”

A correction has been made to the section **1 Introduction**, Paragraph 3:

“There are significant differences between China and foreign countries in terms of system design and operational efficiency of pension insurance funds. In the face of the pressure of aging, pension insurance systems of the United Kingdom, the United States and other countries have adopted two main reform measures. One is to adjust the existing system to balance income and expenditure by reducing pensions or extending the calculation period and delaying retirement. The other is the capitalization transformation, which promotes the implementation of private pension schemes and supports a hybrid model through state guarantees and tax incentives (8). The efficient management and long-term investment capacity of the fund can not only alleviate fiscal pressures on governments, but also have a positive impact on economic growth and social cohesion by promoting long-term savings, promoting employment, and enhancing personal financial responsibility (5). As an important pillar of the welfare state, public pension insurance funds ensure the financial security of the older adult group after retirement and reduce the risk of poverty (9). At the same time, the optimization of fund governance and investment strategies is crucial to improving operational efficiency. The United Kingdom and the United States maintain pension systems through a pay-as-you-go model, but their effectiveness relies on the premise that population growth is higher than interest rates (10). Compared with these countries, the management of China's pension insurance fund faces greater challenges in cross-regional and cross-system coordination. These regional differences provide a practical basis for examining the operational efficiency of URRSEI from a regional perspective. China needs to explore a more flexible reform path to ensure the long-term sustainability of pension funds, promote policy innovation and optimize fund management. While ensuring fairness, the efficient operation of the fund can further enhance the social security function and provide a solid foundation for economic transformation and social progress (2, 8). Therefore, it is necessary to evaluate the operational efficiency of URRSEI in China (11). How to accurately assess the efficiency of its operation has become a very important issue.”

A correction has been made to the **section 2 Literature review**, *subsection 2.1 Efficiency evaluation of URRSEI*, Paragraph 2:

“There are significant differences in the operational efficiency of the pension system between different regions. Although the overall operational efficiency of China's pension insurance is at a high level, there are significant differences in performance between regions (1). Economically developed regions do not necessarily have higher pension performance, and there is not a complete coordination between the level of economic development and pension insurance performance in each province (18). The rural–urban divide is also an important issue, with the rural population's pension insurance participation rate much lower than that of the urban population, especially among migrant workers in the construction industry, many of whom are unable to participate in urban pension schemes due to policy inflexibility (19, 20). The impact of pension on the older adult aged 60–69 years, those with low educational attainment, and the older adult in the eastern region is more significant (21). In addition, the coverage and development level of pension insurance between regions also showed a trend of “high in the east and low in the west,” and the coordination between the economic development level and pension insurance in each province was insufficient (18). There is a significant gap in the operational efficiency of URRSEI in different regions, which reveals the constraints of the lag in policy formulation on the coordinated development of the region. The empirical analysis therefore focuses on how fiscal input, demographic pressure, and regional development conditions are associated with URRSEI efficiency. From 2016 to 2020, the performance of China's urban workers' pension insurance showed a downward trend in various provinces, and regional development was uneven and uncoordinated (18). The existence of such regional differences means that to improve the efficiency of URRSEI, it is necessary to optimize the policy design and promote the coordinated development of various regions.”

A correction has been made to the section **3 Methodology**, Paragraph 4:

“In this paper, two key input indicators are chosen to comprehensively assess the URRSEI system. The income of URRSEI is selected as one of the input indicators. The income of the fund serves as a fundamental measure of the economic foundation and the extent of financial support for URRSEI. It is used to quantify the influx of funds into the system. A higher income indicates a more substantial financial base, which can potentially support better pension provisions and system operations. For example, if the income mainly comes from contributions by insured individuals and government subsidies, a larger income implies more resources available for paying out pensions in the future. Without sufficient income, the system may struggle to meet its long-term obligations, making it a crucial indicator for understanding the system's financial viability. The number of people insured by URRSEI is the second input indicator. This reflects the participation level of residents in the URRSEI program. A larger number of insured people not only broadens the financial base through contributions but also indicates a higher degree of social coverage. When more people are insured, it implies that the system is fulfilling its social security function more effectively, reaching a wider segment of the population. This is significant as the goal of URRSEI is to provide a safety net for as many urban and rural residents as possible. If the number of insured people is declining, it may signal potential problems such as low awareness of the program, unaffordable contribution levels, or lack of trust in the system (42).”

A correction has been made to the References section: References 4 and 12 have been removed from the reference list, and the remaining references have been renumbered sequentially.

The original version of this article has been updated.

